# Localized Modification of Water Molecule Transport After Focused Ultrasound-Induced Blood–Brain Barrier Disruption in Rat Brain

**DOI:** 10.3389/fnins.2021.685977

**Published:** 2021-07-29

**Authors:** Mun Han, Hyeon Seo, Hyojin Choi, Eun-Hee Lee, Juyoung Park

**Affiliations:** Medical Device Development Center, Daegu-Gyeongbuk Medical Innovation Foundation, Daegu, South Korea

**Keywords:** water transport, aquaporin-4, diffusion tensor imaging, focused ultrasound, blood–brain barrier

## Abstract

Interstitial solutes can be removed by various overlapping clearance systems, including blood–brain barrier (BBB) transport and glymphatic clearance. Recently, focused ultrasound (FUS)-induced BBB disruption (BBBD) has been applied to visualize glymphatic transport. Despite evidence that FUS–BBBD might facilitate glymphatic transport, the nature of fluid movement within the sonication region is yet to be determined. In this study, we sought to determine whether FUS–BBBD may facilitate the local movement of water molecules. Two different FUS conditions (0.60–0.65 MPa and 0.75–0.80 MPa) were used to induce BBBD in the caudate-putamen and thalamus regions of healthy Sprague–Dawley rats. The water diffusion caused by FUS–BBBD was analyzed using the apparent diffusion coefficient (ADC), axial diffusivity, radial diffusivity (RD), and fractional anisotropy, obtained at 5 min, 24 and 48 h, as well as the water channel expression of aquaporin-4 (AQP-4) immunostaining at 48 h after FUS-induced BBBD. In addition, hematoxylin and eosin histopathology and Fluoro-Jade C (FJC) immunostaining were performed to analyze brain damage. The signal changes in ADC and RD in the sonication groups showed significant and transient reduction at 5 min, with subsequent increases at 24 and 48 h after FUS-induced BBBD. When we applied higher sonication conditions, the ADC and RD showed enhancement until 48 h, and became comparable to contralateral values at 72 h. AQP-4 expression was upregulated after FUS-induced BBBD in both sonication conditions at 48 h. The results of this study provide preliminary evidence on how mechanical forces from FUS alter water dynamics through diffusion tensor imaging (DTI) measures and AQP4 expression.

## Introduction

The blood–brain barrier (BBB) is one of the most complex and selective barriers in the human organism and comprises brain-specific endothelial cells connected by tight junctions, which limit the passage of molecules into or out of the brain interstitium. Endothelial cells require contact with various central nervous system cells to establish BBB characteristics; thus, they function within the multicellular neurovascular unit, together with surrounding pericytes, astrocytes, and neurons. The impermeability of the BBB is a major barrier that prevents the accumulation of foreign toxic substances and therapeutic agents in the brain (Pardridge, 2005; Liebner et al., 2018; Saint-Pol et al., 2020; Zhang et al., 2020). Focused ultrasound (FUS) has great potential for delivering therapeutics to the brain without surgical procedures. FUS can selectively and transiently disrupt the BBB in targeted local brain areas through mechanical stress and oscillation of intravenously injected microbubbles. Localized BBB disruption (BBBD) in the brain using FUS combined with microbubbles has been validated by various methods, including magnetic resonance (MR), fluorescence imaging, and acoustic signal analysis (Choi et al., 2009; Baseri et al., 2010; Chen and Konofagou, 2014; Cho et al., 2016; Cheng et al., 2019), and its feasibility and safety have been observed in small-to-large animals (Blackmore et al., 2018; McMahon et al., 2019). In addition to drug delivery, transient and targeted BBBD using FUS can induce biological changes in localized brain areas (Todd et al., 2020). For example, FUS–BBBD itself has been shown to reduce the accumulation of amyloid β (Aβ) and hyperphosphorylated tau, which are thought to be pathogenic in Alzheimer’s disease, without the administration of exogenous therapeutics in the Alzheimer’s disease model (Jordao et al., 2013; Burgess et al., 2014; Leinenga and Götz, 2015; Karakatsani et al., 2019; Pandit et al., 2019).

The clearance system includes metabolism, BBB transport, and non-selective, perivascular efflux (Hladky and Barrand, 2017). The glymphatic system is a recently discovered waste drainage system in the brain that is characterized by cerebrospinal fluid (CSF) influx along the perivascular spaces surrounding the penetrating arteries (Iliff et al., 2012; Benveniste and Nedergaard, 2015). Hypothetically, astroglial water transport has been proposed to support perivascular fluid and solute clearance through aquaporin-4 (AQP-4) water channels, which are localized primarily to perivascular astrocytic endfeet ensheathing the cerebral vasculature (Iliff et al., 2012, 2013; Nedergaard, 2013). Although the biophysical basis of AQP4 in perivascular transport remains unresolved, recent findings on the density of endfoot ensheathment of the vasculature have led to an improved understanding of the role of AQP4 and astroglial endfoot solute transport (Korogod et al., 2015; Iliff and Simon, 2019). This brain-wide glymphatic system facilitates the clearance of interstitial solutes, such as Aβ and tau (Iliff et al., 2012, 2014; Boespflug and Iliff, 2018), and BBB transport has a similar purpose in clearing interstitial solutes (Verheggen et al., 2018). The reduction of Aβ and tau has also been shown by FUS–BBBD with activation of astrocytes and microglia (Jordao et al., 2013; Leinenga and Götz, 2015; Nisbet et al., 2017; Pandit et al., 2019), which implies the possibility of an impact of FUS–BBBD on waste clearance (Todd et al., 2020). A recent study observed that FUS-induced BBBD might drive interstitial flow in the perivascular region (Meng et al., 2019; Curley et al., 2020; Lee et al., 2020). Curley et al. (2020) developed a non-viral gene delivery approach for transfecting brain tumors using FUS with microbubbles and demonstrated interstitial fluid flow changes in response to BBBD. Meng et al. (2019) showed that the MR contrast agent can be delivered to the brain parenchyma, and further observed clearance in the interstitial fluid pathway through the perivenular and subarachnoid spaces, as well as around draining veins. Lee et al. (2020) demonstrated that FUS–BBBD enhances the glymphatic clearance of Aβ mainly by increasing brain-to-CSF drainage. This glymphatic dynamic may be related to biological effects induced by mechanical forces; however, the subsequent changes in water dynamics in the local perivascular region are yet to be clearly established (Burgess et al., 2012; Meng et al., 2019).

Here, we assumed that FUS-induced BBBD would have an influence on the brain-wide clearance system based on previous evidence (Meng et al., 2019; Curley et al., 2020; Lee et al., 2020) and aimed to establish subsequent changes in local water dynamics by FUS-induced BBBD. One method for non-invasive measurement of water diffusion is diffusion tensor imaging (DTI), which is a magnetic resonance imaging (MRI) technique that measures the mobility of water molecules in tissues based on its ability to determine the orientation and diffusion characteristics (Tae et al., 2018). DTI analyzes the characteristics of tissue structure on a microscopic scale through an extensive description of water diffusion by analyzing fractional anisotropy (FA), which describes the orientation coherence of diffusion and the level of structural integrity of the tissue (Baser, 1995; Beaulieu, 2002; Le Bihan, 2003), and apparent diffusion coefficient (ADC), which represents the overall mean diffusivity (Le Bihan, 2003). Because of its non-invasiveness, Taoka et al. (2017) evaluated glymphatic dysfunction in patients with Alzheimer’s disease through water diffusivity along the perivascular space using DTI and showed a positive correlation with the degree of severity using the Mini-Mental State Examination score. In addition, recent studies using DTI have been introduced to evaluate glymphatic function, with a focus on the perivascular fluid (Harrison et al., 2018; Sepehrband et al., 2019; Taoka and Naganawa, 2020).

In this study, we investigated the impact of FUS-induced BBBD on the transport of water molecules in the local sonication region using DTI-MRI and immunochemistry. We induced two BBBD conditions (0.60–0.65 MPa and 0.75–0.80 MPa) to compare the transport phenomenon of water molecules and analyzed the DTI parameters of ADC, axial diffusivity (AD), radial diffusivity (RD), and FA values, at different times (5 min, 24 h, and 48 h) after the FUS-induced BBBD. Furthermore, expression of AQP-4, which supports perivascular fluid and solute movement along the glymphatic system (Thrane et al., 2014; Smith et al., 2017), was investigated at 48 h after FUS-induced BBBD, to examine the molecular changes. Higher sonication conditions were considered to examine whether a higher degree of BBBD was correlated with the degree of water dynamics. Tissue damage was observed using hematoxylin and eosin (H&E) and Fluoro-Jade C (FJC).

## Materials and Methods

### Study Subjects

Twenty-eight male Sprague–Dawley (SD) rats (8 weeks old and weighing 330 ± 28 g, Orient Bio Inc., Seongnam, South Korea) were used. This study was approved by the Institutional Animal Care and Use Committee of the Daegu Gyeongbuk Medical Innovation Foundation. Two rats were housed in cages at 20–25°C with a 12 h light/dark cycle. All procedures and handling of the animals were performed according to the ethical guidelines for animal studies.

### BBBD System

**FIGURE 1:**
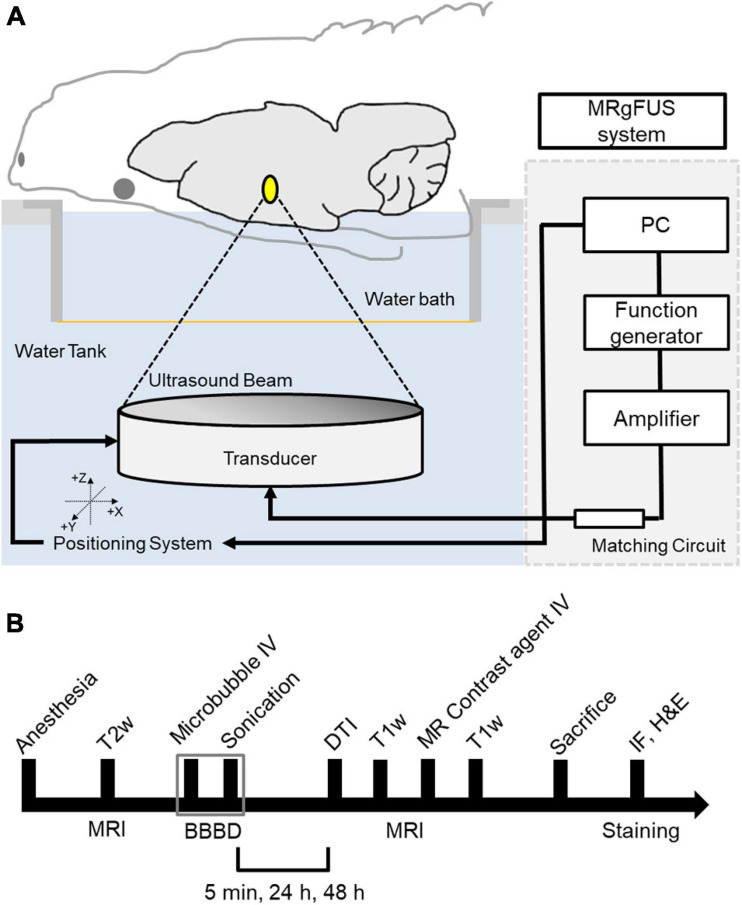
Schematic of the MRI-guided FUS system and experimental design. **(A)** The animal is in supine position with its head submerged in water tank. The focal area is targeted with MR image guidance and PC-controlled positioning system. **(B)** Experimental design for investigating the effects of FUS–BBBD on water transport. T2-weighted MR images were obtained to locate the focal region before the sonication, and T1-weighted images and diffusion tensor images were acquired for each time slot (5 min, 24 and 48 h) after the FUS–BBBD. Sprague–Dawley rats were sacrificed and perfused for staining after 48 h after the sonication.

The MRI-guided focused ultrasound (MRgFUS) system (RK-100, FUS instruments, Toronto, Canada) was used to sonicate rat brains for BBBD, as described previous study (Cho et al., 2016). A schematic of the system is shown in Figure 1A. This system uses a single-element therapeutic FUS transducer (diameter: 75 mm; radius: 60 mm; center frequency: 1.1 MHz) to generate ultrasound. The FUS pressure distribution at the focal region was measured in a free water field using an acoustic intensity measurement system (AIMS III, ONDA, Sunnyvale, CA, United States) and a hydrophone (HGL-400, ONDA, Sunnyvale, CA, United States). The system was attached to a computer-controlled three-dimensional positioning system. The transducer was submerged in a water tank filled with degassed water, and the animal was placed in a supine position on an MR-compatible animal bed with its head partially submerged in water. MRI was utilized as an image guidance for the MRgFUS system. MR images were used to target sonication in specific regions of the rat brain. The images taken from the MRI were transferred to the MRgFUS system, and the coordinates were synchronized between the two systems.

### BBBD Experiments

The procedure for FUS-induced BBBD was performed according to a previously described method (Cho et al., 2016) with the addition of DTI acquisition after BBBD to detect water diffusivity. The animals were anesthetized intramuscularly with a mixture of Zoletil 25 mg/kg (Virbac Laboratories, Carros, France) and Rumpun (4.6 mg/kg; Bayer, Leverkusen, Germany), and were constantly monitored throughout the experimental procedures to ensure no evidence of pain or suffering. Hair was removed from the head using a shaving razor and hair-removal cream. The animals were placed in a supine position on an MR-compatible animal bed. The experimental procedure is illustrated in Figure 1B.

The BBBD target regions were the caudate putamen (CP) and thalamus (TH). Before sonication, the microbubbles (0.02 mL/kg, DeFinity, Lantheus Medical Imaging, North Billerica, MA, United States) were diluted in a ratio of 1:50 in normal saline, and then injected through the tail vein catheter using an automated syringe pump (Pump 11, Harvard Apparatus, Holliston, MA, United States) for 10 s as an initiation. This was performed to ensure that the circulating microbubbles fully reached the target region. Thereafter, 0.60–0.65 MPa or 0.75–0.80 MPa acoustic pressure was applied at the target regions (CP and TH) to induce FUS–BBBD with the diluted microbubbles. Microbubbles were infused for more than 90 s. The FUS energy was delivered with pulsed sonication consisting of 10 ms tone bursts at a pulse repetition frequency of 1 Hz for 120 s. After the BBBD, the DTI was acquired at 5 min, 24 h, and 48 h. T1-weighted MR images were obtained with a 0.2 mM/kg gadolinium-based contrast agent (Dotarem, Guerbet, Roissy, France) to confirm the BBBD. All rat brains were perfused and fixed through transcardial perfusion (0.9% normal saline, 200 mL; 4% buffered formalin phosphate, 250 mL) at each time point. Thereafter, the brains were harvested and processed for AQP-4 staining, H&E staining, and FJC staining.

### Study Design

Sprague–Dawley rats were randomly assigned to three groups according to the study design, as summarized in Table 1. Two sonication groups and a sham-controlled group were determined using MR signal analysis. Based on earlier studies, different degrees of BBBD were found depending on the brain region, resulting in smaller BBB permeability in CP compared to TH (Huh et al., 2020); thus, we experimentally determined how to apply a higher acoustic power to CP than to TH, such that the target regions showed comparable degrees of BBBD. We designated the sonication groups as group-0.65 MPa and group-0.80 MPa, which indicated the corresponding acoustic power applied to CP.

**TABLE 1:** Summary of experimental FUS parameters.

**Group**	**Acoustic pressure (MPa)**	
	**Caudate-putamen (CP)**	**Thalamus (TH)**
Group-Sham	0	0
Group-0.65 MPa	0.65	0.60
Group-0.80 MPa	0.80	0.75

Twenty-eight male SD rats were used in this study, as summarized in Table 2. The first eight SD rats were used in a pilot study to define the experimental settings for the DTI image optimization, FUS parameters for BBBD conditions, and AQP-4 and FJC staining tests. Eight rats were used in the sonication group. In the group-0.65, five rats were used, although a follow-up analysis was not performed on two rats due to an insufficient degree of BBBD. Three rats were assigned to group-0.80 MPa and group-sham, respectively. For the AQP-4 evaluation, five rats were used; four rats were used for H&E histology and FJC staining to assess damage inflicted on the rat brain.

**TABLE 2:** Summary of animal numbers.

**Experiments**	**Pilot study**	**MRI scan (DTI measures)**	**AQP-4**	**Histopathology (H&E and FJC)**
Animal number	8	11	5	4

### MR Imaging

Imaging was performed using a 9.4-T preclinical MRI system (BioSpec 94/20 USR, Bruker, Ettlingen, Germany). T2-weighted images were used to select sonication targets prior to FUS treatment. T1-weighted contrast-enhanced images were used to evaluate the BBBD. DTI was used to evaluate the diffusion of the water molecules. The following MRI parameters were employed for 2D rapid acquisition with refocused echoes-T1-weighted images: field of view = 30 mm × 30 mm, matrix size = 256 × 256, axial slices = 26, coronal slices = 10, axial slice thickness = 1.0 mm, coronal slice thickness = 1.5 mm, slice gap = 0, repetition time (TR) = 1,500 ms, echo time (TE) = 6.5 ms, and number of averages = 3; T2-weighted images: TR = 2,500 ms, TE = 33 ms, number of averages = 2, and the other parameters were equal to those of the T1-weighted images. Single-shot spin-echo planar imaging was used to obtain the DTI images. The DTI parameters were as follows: field of view, 30 mm × 30 mm; matrix size, 128 × 128; axial slices, 26; slice thickness, 1.0 mm, with no gap; TR = 2,000 ms; TE = 26 ms; number of averages = 2; *b*-value = 1,000 s/mm^2^; and diffusion direction = 30. DTI images were obtained before injecting the MR contrast agent to avoid imaging the effect of the contrast agent. During the MRI scans, the temperature of the animals was maintained at approximately 37°C using a warm water blanket.

### MR Data Analysis

**FIGURE 2:**
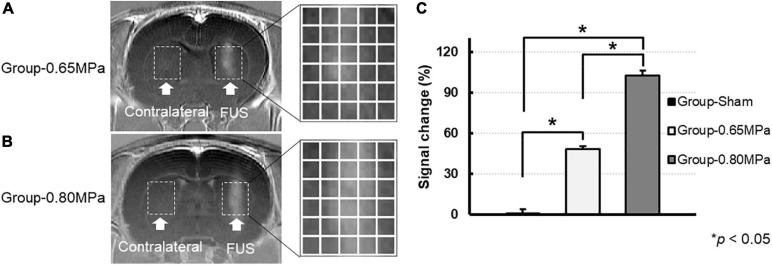
Magnitude of BBBD according to the MR signal change. T1-weighted contrast-enhanced images 5 min after the FUS were used to verify the opening of the BBB for group-0.65 MPa **(A)** and group-0.80 MPa **(B)**. The white dotted lines represent the ROIs in sonication (FUS) and contralateral regions **(C)**. The enhanced signal after the FUS sonication in ROIs accessed by each group. *p < 0.05.

In order to normalize the slight intensity differences between the images, all T1-weighted images were normalized using background noise signal intensity before analysis. To confirm the degree of BBBD in the T1-weighted images, T1-weighted contrast-enhanced images were subtracted from the free contrast agent T1-weighted images. The rectangular region of interest (ROI) in the FUS treatment region and contralateral region was manually outlined (Figures 2A,B; white dotted line). The ROIs were divided into 5 × 6 regions and used for the analysis. The change in MR signal intensity in T1-weighted images is defined as follows:

(1) $$SignalChange\left(\%\right)=\left(\frac{{\text{SI}}_{\text{A}}-{\text{SI}}_{\text{B}}}{{\text{SI}}_{\text{B}}}\right)\times 100$$

where SI_A_ and SI_B_ are the signal intensities in the FUS sonication and contralateral regions, respectively.

The DTI data were analyzed using the Diffusion Toolkit (Wang et al., 2007), as shown in Supplementary Figure 1. DTI was performed with 30 different diffusion gradient directions. For raw DTI data processing, S_ij_(b)/S_i_(0) = exp(-bD), where S_ij_(b) is the signal intensity of pixel I, with a diffusion gradient in the direction j, and S_i_(0) is the signal intensity of the same pixel at *b* = 0 mm^2^s. This fitting minimized the sum of the squares of the 30 non-linear functions for each diffusion gradient direction in the six diffusion tensor variable components by modifying the Levenberg–Marquardt algorithm (Marquardt, 1963). The diffusion tensor was diagonalized by applying a principal component analysis (Jolliffe, 1990) to yield the major (λ_1_), intermediate (λ_2_), and minor (λ_3_) eigenvalues corresponding to the three eigenvectors in the diffusion tensor matrix. The DTI measures of ADC, AD, RD, and FA were calculated using the following formula:

(2) $$ADC=\frac{\lambda_{1}+\lambda_{2}+\lambda_{3}}{3},AD=\lambda_{1},RD=\frac{\lambda_{2}+\lambda_{3}}{2}$$

(3) $$FA=\frac{1}{\sqrt{2}}\sqrt{\frac{{\left(\lambda_{1}-\lambda_{2}\right)}^{2}+{\left(\lambda_{2}-\lambda_{3}\right)}^{2}+{\left(\lambda_{1}-\lambda_{3}\right)}^{2}}{\lambda_{1}^{2}+\lambda_{2}^{2}+\lambda_{3}^{2}}}$$

For quantitative evaluations, software supplied by MATLAB v9.0 (The Mathworks Inc., Natick, MA, United States) was used to determine the signal intensity, which is defined as the mean of the ADC and FA signal intensities. The same ROIs in the T1-weighted images were used for all the analyses.

### Immunofluorescence (AQP-4 Staining)

Sprague–Dawley rats were sacrificed 48 h after BBBD. Immunofluorescence was performed as described previously (Choi et al., 2019). Briefly, the brains of the rats were transcardially perfused with 0.9% sodium chloride (NaCl) followed by post-fixation overnight in ice-cold 4% formaldehyde. The fixed brains were dehydrated using 10%, 20%, and 30% sucrose gradients for cryoprotection. The frozen brain tissue was cut into 50-μm thick slices using a cryostat (CM1860, Leica, Nussloch, Germany), and tissue slices were permeabilized for 30 min in 0.2% Triton X-100 (Sigma-Aldrich, St. Louis, MO, United States). The permeabilized tissues were incubated for 2 h in a blocking solution containing 10% normal goat serum (Abcam, Cambridge, MA, United States), followed by overnight incubation with a rabbit monoclonal anti AQP-4 (Cat No. ab9512 1:250; Abcam, Cambridge, MA, United States). Subsequently, the slices were incubated with Alexa Fluor 488-conjugated mouse anti-rat IgG (Abcam, Cambridge, MA, United States) for 1 h at room temperature. The slides were mounted using a fluorescence mounting medium (Dako, Glostrup, Denmark). The tissue slides were scanned using a slide scanner (Pannoramic Scan II, 3D Histech, Budapest, Hungary), and the acquired images were processed using CaseViewer software (2.1v, 3D Histech, Budapest, Hungary). To quantify the AQP-4 values, we performed manual co-registration of AQP-4 and MR images to have comparable structures using MATLAB and analyzed AQP-4 values in the same ROIs as those in the MR image analysis.

Furthermore, we performed immunohistochemistry for the glial fibrillary acidic protein (GFAP) and AQP-4. The slides were rescanned by Axio Scan.Z1 microscope (Carl Zeiss, Göttingen, Germany) for the acquisition of high-resolution images. A magnified astrocyte was co-labeled with GFAP (red), AQP-4 (green), and DAPI (blue). The acquired images were post-processed using the Zen 2 image-processing software (blue edition, Carl Zeiss) and adjusted the values for brightness (white), gamma, and contrast (black) to reduce background noise. We applied the same parameters for all slides in both sonication and contralateral hemisphere.

### Histology

For H&E staining, the SD rats were sacrificed 48 h after BBBD. The harvested brains were embedded in paraffin blocks and serially sectioned at 5-μm thickness in the axial plane. H&E staining was performed every 50th section (250 μm apart) using an H&E staining kit (VECTOR Laboratories, Burlingame, CA, United States). Images were recorded using a slide scanner (Pannoramic Scan II, 3D Histech, Budapest, Hungary), and the area of red blood cells in the sonicated brain region was observed using Image J (National Institutes of Health, Bethesda, MD, United States).

### Fluoro-Jade C Staining

A FJC staining kit (Biosensis Inc., Thebarton, SA, Australia) was used to observe neuron degeneration by the FUS–BBBD and was applied to the cryosections according to the manufacturer’s instructions. The slides were immersed in a 10% sodium hydroxide solution (v/v) for permeabilization, followed by incubation in 10% potassium permanganate solution (v/v) for blocking. The slides were transferred into a mixture of 20% FJC and 20% 4,6-diamidino-2-phenylindole (DAPI) (v/v), which was dissolved in 0.1% acetic acid. The slides were dried and covered with coverslips using dibutylphthalate polystyrene xylene (DPX) mounting medium (Sigma-Aldrich, St. Louis, MO, United States).

### Statistical Analyses

Statistical analyses were performed using the commercial software (IBM Statistical Package for the Social Sciences 21.0, IBM Corp., Armonk, NY, United States). We presented the average data of two target regions (CP and TH) as the mean ± standard error. The sham-controlled and sonication groups (group-0.65 MPa and group-0.80 MPa, respectively) were compared via the Kruskal–Wallis one-way analysis of variance (ANOVA) on ranks with *post hoc* Tukey’s HSD test in terms of the MR signal change in the T1-weighted images, ADC, AD, RD, FA, and AQP-4 values. The significance of the difference was considered statistically significant at *p* < 0.05.

## Results

### FUS-Induced BBBD

In this study, we induced BBBD with two FUS parameters (0.60–0.65 MPa and 0.75–0.80 MPa) to compare the water diffusion. The FUS-induced BBBD at the targeted brain regions was confirmed using T1-weighted contrast-enhanced images at 5 min after BBBD (Figure 2). The MR signal intensity at both the sonication conditions was significantly increased when compared with the sham control regions, and the degrees of BBBD in group-0.80 MPa showed significant enhancement when compared with group-0.65 MPa (Figure 2C). The signal intensity changes in the ROI in the BBBD region, when compared with the contralateral region of the T1-weighted images, were 0.88 ± 1.0%, 48.6 ± 6.2%, 102.7 ± 7.4% in group-sham, group-0.65 MPa, and group-0.80 MPa, respectively (Figure 2C).

### Water Transport Analysis After FUS-Induced BBBD

**FIGURE 3:**
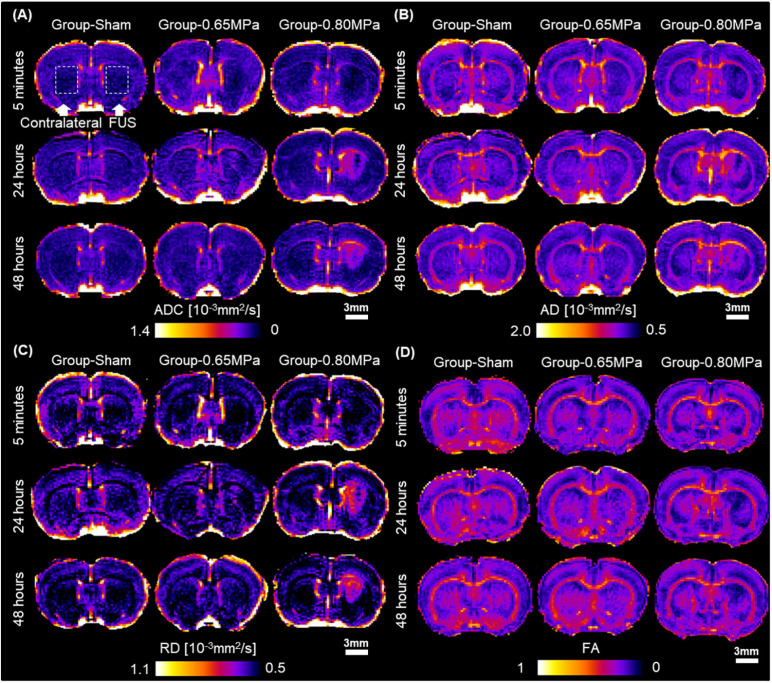
DTI measures after FUS–BBBD. **(A)** Representative maps of apparent diffusion coefficient (ADC), **(B)** axial diffusivity (AD), **(C)** radial diffusivity (RD), and **(D)** fractional anisotropy (FA) at caudate putamen. Scale bar: 3 mm.

For the quantitative measurement of water diffusivity according to the FUS treatment conditions, we obtained the diffusion tensor to derive the ADC, AD, RD, and FA values. Figure 3 shows representative maps of ADC, AD, RD, and FA values after FUS–BBBD.

On ADC maps, a decreased signal in sonicated regions was observed in sonication groups at 5 min, and a higher signal in the sonicated regions compared with those in the contralateral regions was observed in group-0.80 MPa after 24 and 48 h. Consistent with ADC maps, this difference between groups and times was clearly observed in the AD and RD maps. In the FA maps, a reduced signal was consistently observed in the sonicated groups.

**FIGURE 4:**
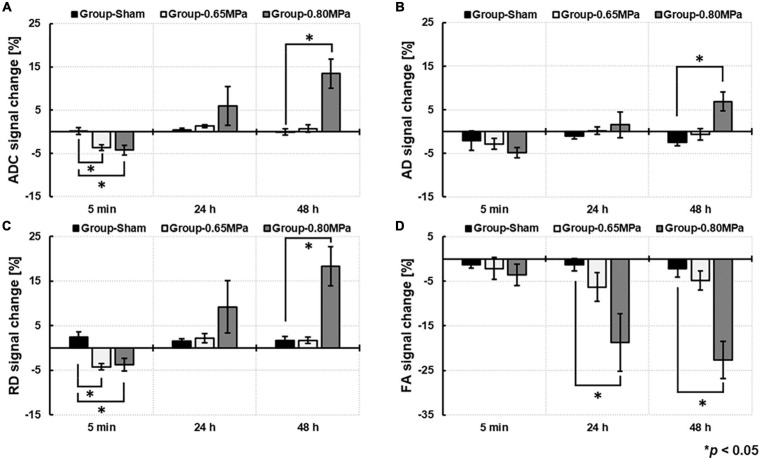
Comparison of DTI measures of **(A)** ADC, **(B)**, AD, **(C)** RD, and **(D)** FA based on the signal change ratio between group-sham, group-0.65 MPa, and group-0.80 MPa after 5 min, 24 and 48 h of the FUS–BBBD. ^∗^*p* < 0.05.

For the statistical analyses, we used signal changes of ADC, AD, RD, and FA by the ratio of the values in the ROIs in the sonicated region to those in the contralateral ROIs (Figure 4).

At 5 min after the FUS–BBBD, the sonication groups showed a decrease in signal changes for ADC and RD. After 24 h, the signal changes of ADC (1.0132 ± 0.0034) and RD (1.0219 ± 0.0108) showed a slight increase in group-0.65 MPa and a relatively higher increase in group-0.80 MPa (ADC: 1.0595 ± 0.0447; RD: 1.0919 ± 0.0582); however, they showed no statistical significance when compared with that of group-sham (ADC: 1.0045 ± 0.0072; RD: 1.0155 ± 0.0048). A significant decrease in FA signal changes (−6.3135 ± 3.5691) was calculated for group-0.80 MPa at 24 h when compared with that of the sham group (−1.3030 ± 1.4575). After 48 h, parametric diffusion (ADC, AD, and RD) showed no significant difference between group-0.65 MPa (ADC: 1.0073 ± 0.0082; AD: 0.9941 ± 0.0132; RD: 1.0171 ± 0.0070) and group-sham (ADC: 0.9991 ± 0.0072; AD: 0.9757 ± 0.0083; RD: 1.0167 ± 0.0095), and there was an increase in group-0.80 MPa (ADC: 1.1345 ± 0.0339; AD: 1.0690 ± 0.0218; RD: 1.1830 ± 0.0443) when compared with that of the group-sham (*p* < 0.05). In group-0.80 MPa, a continuous FA decrease (−22.6496 ± 4.5203) was observed, resulting in significant differences compared with group-sham (−2.1775 ± 2.0283) and group-0.65 MPa (−4.7937 ± 2.3636) at 48 h after the FUS–BBBD.

**FIGURE 5:**
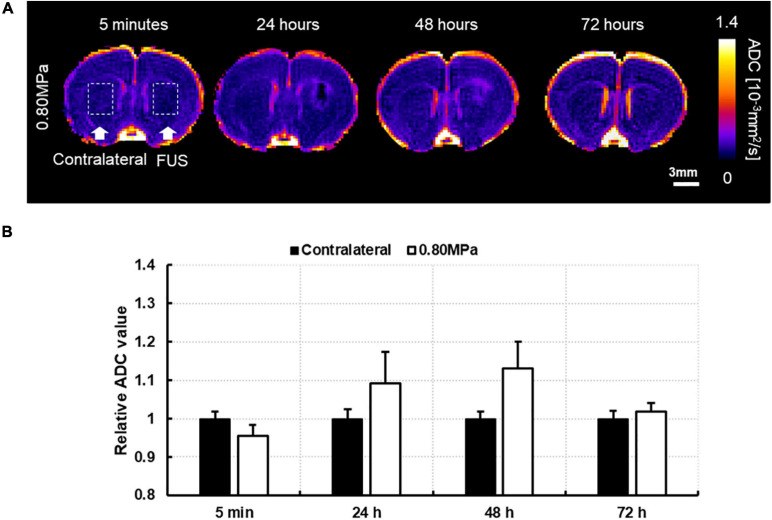
**(A)** A representative image of follow-up ADC at 5 min, 24, 48, and 72 h after 0.80 MPa sonication at caudate putamen. **(B)** The ADC signals in ROI are analyzed in sonication and contralateral region.

Owing to the significant increase in DTI measurements at 48 h in group-0.80 MPa, we attempted to observe the signal changes of ADC at 72 h from a randomly selected case in group-0.80 MPa to determine whether the ADC increase in group-0.80 MPa was persistent or not. As shown in Figure 5, the ADC value in the sonication region was comparable with the contralateral region at 72 h, indicating transient signal changes after FUS exposure in group-0.80 MPa.

**FIGURE 6:**
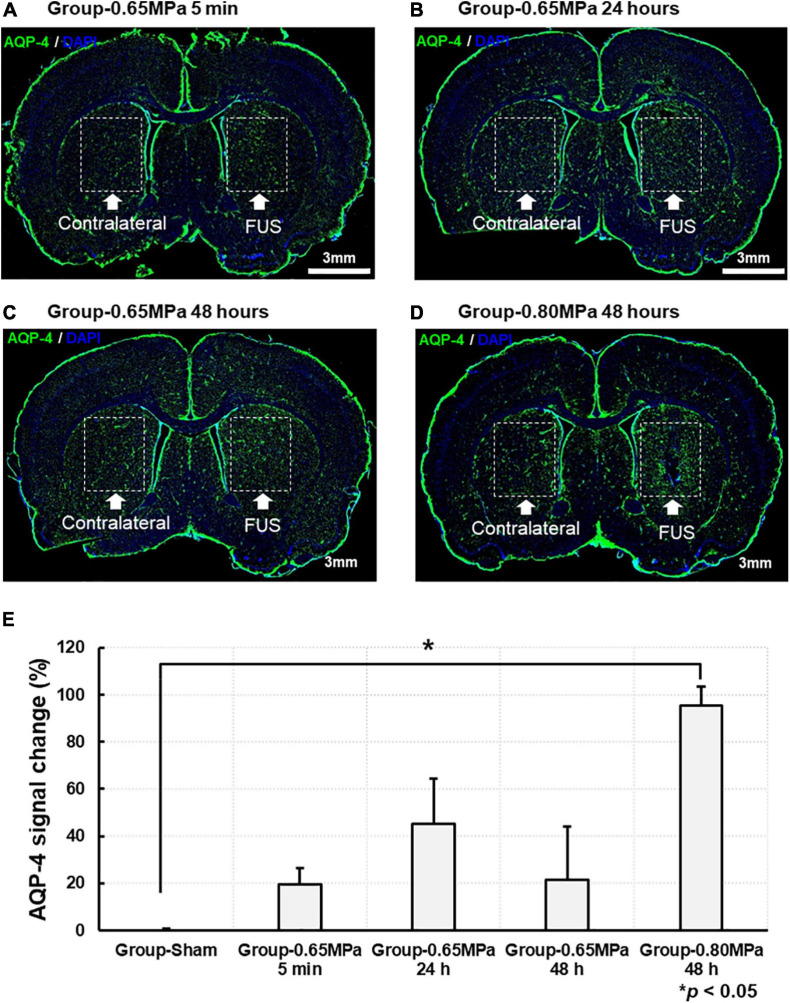
Representative AQP-4 immunostaining at caudate putamen for group-0.65 MPa and group-0.80 MPa. The brain section was stained with AQP-4 for group-0.65 MPa at 5 min **(A)**, 24 h **(B)**, and 48 h **(C)** and group-0.80 MPa at 48 h **(D)** after sonication. Green and blue fluorescence indicate the AQP-4 expression and the DAPI, respectively. Scale bar: 3 mm. **(E)** Comparison of the AQP-4 signal change is analyzed between group-sham, group-0.65 MPa, and group-0.80 MPa. *p < 0.05.

Changes in AQP-4 expression are shown in Figure 6. The AQP-4 signal changes in the same ROIs as the MRI analyses were calculated in the FUS sonicated region, compared with those of the contralateral region, and statistically analyzed in each group. We characterized AQP-4 expression at different time points after sonication in group-0.65 MPa. Where significance was not found, AQP-4 upregulation continued for 48 h, peaking at 24 h. Then, we evaluated changes in AQP-4 expression at 48 h after the FUS–BBBD, as significant changes were observed in all DTI measures (ADC, AD, RD, and FA) for group-0.80 MPa. At 48 h, group-0.65 MPa showed a higher AQP-4 signal change (21.411 ± 22.506) than group-sham (−0.556 ± 1.374), although no statistically significant differences were observed (*p* > 0.05). In group-0.80 MPa, the AQP-4 signal change was significantly increased (95.513 ± 7.832) when compared with that in group-sham (−0.556 ± 1.374, *p* < 0.05) at 48 h.

**FIGURE 7:**
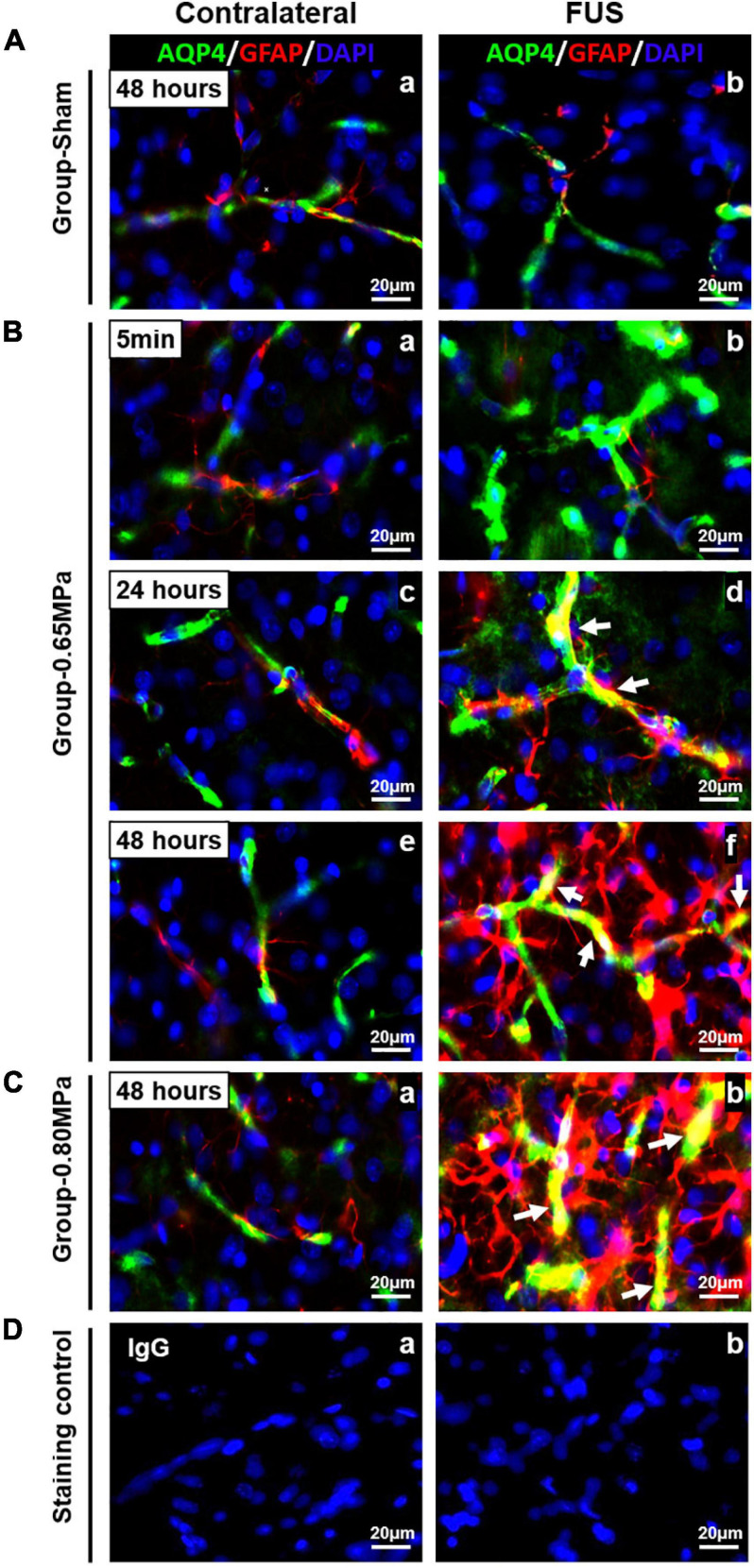
Co-localization of AQP-4 astrocytic endfeet and GFAP in the FUS–BBBD region. Immunofluorescence staining for GFAP-positive astrocytes (red) and AQP astrocytic endfeet [green was performed in brain tissue for group-sham **(A)**, group-0.65 MPa **(B)**, group-0.80 MPa **(C)**, and staining control **(D)**]. For group-0.65 MPa, we analyzed the changes of AQP-4/GFAP expression at multiple time points after FUS–BBBD. At 5 min, an increase of AQP-4 expression is shown that do not overlap with GFAP staining (Bb). At later time points after sonication, yellow colors in sonication region confirm co-localization of AQP-4 and GFAP (Bd, Bf, and Cb; white arrows). Contralateral regions are shown as a control. Blue fluorescence indicates the DAPI. Scale bar, 20 μm.

In Figure 7, we performed immunohistochemistry for GFAP and AQP-4 to clarify the functional relationship between the juxtavascular astrocytes and the cellular localization of astrocytic endfeet. At 5 min in group-0.65 MPa, AQP-4 expression was highly increased in perivascular region (green) (Figure 7Bb). At 24 h and 48 h, increased AQP-4 expression was remarkably co-localized in juxtavascular astrocytes in group-0.65 MPa (yellow) (Figures 7Bd,f,Cb). At 48 h, group-0.80 MPa showed intensive upregulation of AQP-4 and juxtavascular astrocytes than group-0.65 MPa (Figure 7C). The co-localization of GFAP and AQP-4 demonstrated a dense attachment surrounding blood vessel-like structures. This indicated that FUS–BBBD induced an increase in AQP-4 subcellular localization to the BBB through structural changes in astrocytic endfeet.

### Histology and Fluoro-Jade C

**FIGURE 8:**
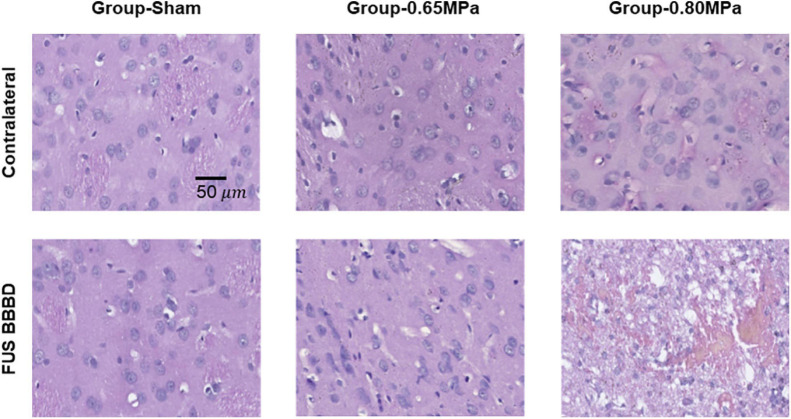
Representative H&E histology at 48 h after the FUS–BBBD. The samples of group-0.65 MPa show no apparent tissue damage, while the samples of group-0.80 MPa show extravasation of red blood cells. Scale bar of H&E: 50 μm.

Figure 8 shows representative samples of the histologic evaluation of H&E stained sections.

**FIGURE 9:**
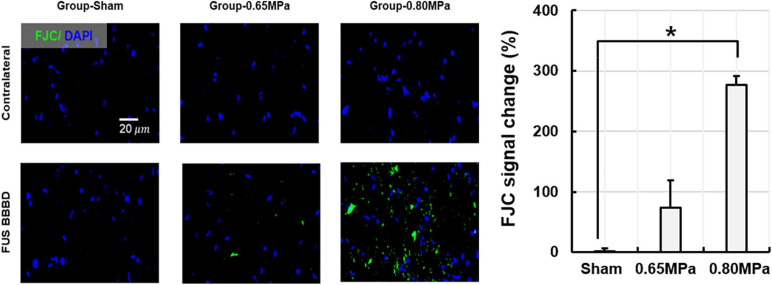
Representative Fluoro-Jade C (FJC) histology and its quantification at 48 h after the FUS–BBBD. The samples of group-0.65 MPa show weakly expressed FJC, while those of group-0.80 MPa show notable FJC expression, indicating neuron degeneration. Green and blue fluorescence indicate the FJC expression and the DAPI, respectively. Scale bar of FJC: 20 μm. *p < 0.05.

In group-0.65 MPa, no apparent change in tissue was observed, although a red blood cell extravasation was induced when higher acoustic pressure was applied. To evaluate neurodegeneration caused by FUS-induced BBBD, FJC staining was performed (Figure 9).

Our results confirmed weak expression of FJC in the sonicated region for group-0.65 MPa, and a notable FJC expression after higher sonication in group-0.80 MPa. Thus, group-0.65 MPa showed no histological damage or mild neurodegeneration, while the higher sonication used in group-0.80 MPa caused histological damage and neurodegeneration.

## Discussion

The glymphatic system is a brain-wide clearance pathway that supports the rapid exchange of CSF and interstitial fluid (ISF) along perivascular pathways, contributing to the efflux of interstitial solutes, including Aβ (Iliff et al., 2012). Interstitial solutes can be removed via various overlapping clearance systems, including BBB transport or glymphatic clearance (Tarasoff-Conway et al., 2015), and there is a possibility of interaction between the BBB and the glymphatic system in clearing interstitial solutes (Verheggen et al., 2018).

Accumulation of Aβ and tau results from an imbalance between its production and clearance, which is the key histopathological hallmark of Alzheimer’s disease, and a defective clearance system can be a possible candidate to explain Alzheimer’s disease development and Aβ accumulation (Verheggen et al., 2018). Previously, the majority of Aβ was known to be cleared across the BBB (Shibata et al., 2000), although recent studies have shown that the glymphatic system is important for Aβ clearance (Iliff et al., 2012; Kress et al., 2014). Although the precise routes and fluid dynamics in glymphatic transport remain controversial, the investigation of the waste clearance system is an attractive alternative for pathological conditions, such as Alzheimer’s disease, as it can remove key proteins involved in neurodegeneration, without a requirement for specific transporters (Mestre et al., 2020).

The primary effect of FUS with microbubbles is drug delivery via targeted and reversible disruption of the BBB, and thus, the exogenous antibodies to reduce Aβ were delivered successfully to the brain (Raymond et al., 2008; Jordão et al., 2010). When FUS is applied without additional drug delivery, several studies have shown a reduction in Aβ and changes in behavior with increased neuronal plasticity in Alzheimer’s disease mouse models (Jordao et al., 2013; Burgess et al., 2014; Leinenga and Götz, 2015). It has been suggested that FUS–BBBD activates astrocytes and microglia and contributes to Aβ internalization and clearance (Jordao et al., 2013). A recent investigation showed a CSF clearance system following FUS-induced BBBD. Meng et al. (2019) examined the MRI images acquired in eight subjects with Alzheimer’s disease and four with amyotrophic lateral sclerosis following FUS–BBBD, and observed clearance patterns of gadobutrol in the perivascular space, subarachnoid space, and around draining veins. Despite evidence that FUS–BBBD might facilitate waste removal, the nature of fluid movement within the perivascular space is yet to be determined (Todd et al., 2020).

The perivascular space is considered a key component of the glymphatic pathway, as it is hypothesized that convective flux from the perivascular space drives interstitial solutes via perivenous channels (Iliff et al., 2012, 2013). To extend the evidence of the glymphatic system that has been investigated by invasive approaches in animal experiments, several studies have recently highlighted the potential of DTI for the non-invasive assessment of perivascular function (Taoka et al., 2017; Harrison et al., 2018; Sepehrband et al., 2019; Taoka and Naganawa, 2020). Taoka et al. (2017) proposed a DTI analysis along the perivascular space based on prior knowledge that major fiber tracts have different directions from the perivascular region at the level of the lateral ventricle body, which was confirmed through high-resolution imaging and color-coded FA images of normal brains. Thus, a histological change that impaired water diffusivity in the direction of the perivascular region would alter diffusivity along the fibers, and this change was related to the Alzheimer’s disease severity. In addition, the perivascular space appears to be the main feature inducing different mean diffusivity between cognitively normal and mild cognitive impairment subjects (Sepehrband et al., 2019). Under normal conditions, the increase in mean diffusivity was investigated between the morning and afternoon and between sleep and wakefulness, suggesting enhanced glymphatic transport (Thomas et al., 2018; Demiral et al., 2019). Together, these studies suggest that diffusion-weighted MRI is a promising non-invasive approach for the evaluation of the glymphatic system.

Despite the evidence of water clearance by FUS–BBBD (Meng et al., 2019; Lee et al., 2020), the water dynamics in the local sonication region have yet to be ascertained. Here, we aimed to explore whether FUS–BBBD could induce local water diffusivity changes, which would provide preliminary support for understanding the underlying mechanisms of waste clearance by FUS–BBBD.

In this study, BBBD was evaluated for two different FUS strengths. The acoustic pressure of FUS was used by 0.60 MPa (TH) and 0.65 MPa (CP) for group-0.65 MPa and by 0.75 MPa (TH) and 0.80 MPa (CP) for group-0.80 MPa, respectively. In an earlier study, different degrees of BBBD were found depending on the brain region, resulting in smaller BBB permeability in CP compared to TH (Huh et al., 2020). Thus, by applying different acoustic pressures, the two target areas showed comparable degrees of BBBD, comparable patterns of DTI parameters, and histological results. Since the FUS parameter in group-0.65 MPa has been widely used in the rat brain to safely disrupt the BBB and deliver therapeutics in several previous studies (Kinoshita et al., 2006b; Cho et al., 2016; Jung et al., 2019), we selected the FUS parameter to determine whether water transport could be facilitated in the BBBD brain area. We additionally applied higher sonication conditions in group-0.80 MPa to evaluate the effect of the magnitude of BBB opening on local water dynamics. As the group-0.80 MPa induced BBBD accompanied by red blood cell extravasation, we examined various FUS spectra covering known biological and pathological conditions. The degree of BBBD was monitored by the change in signal intensity of T1-weighted contrast-enhanced MRI, resulting in a signal change of approximately 50% for group-0.65 MPa and about 100% signal change for group-0.80 MPa because the MR signal intensity has been shown to correlate with the degree of BBBD via dye leakage staining into the brain (Kinoshita et al., 2006a,b; Hsu et al., 2013).

We further performed histological evaluation via H&E and FJC staining to observe tissue damage and neuronal degeneration. In group-0.65 MPa, no tissue damage effects and only a few damaged neurons (FJC-positive cells) were noted in the sonicated samples. At higher acoustic pressures in group-0.80 MPa, we observed a greater increase in the number of extravasated red blood cells, FJC-positive neuronal cells, and microvacuolations in the FUS-BBBD region (Figures 8, 9). In general, FUS–BBBD may undergo acute tissue deterioration according to the FUS intensity. Although group-0.8 MPa induced BBBD accompanied histological tissue damages, we considered that the physiological mild damage induced by 0.8 MPa sonication was insufficient to lead to glioma or necropsy. However, our histological analysis data were limited to the determination of tissue necrosis or astrogliosis. Thus, additional staining (such as TUNEL or glioma-specific marker) will be performed in future studies to fully understand the histological damages.

To explore water dynamics in the local sonication region, we analyzed water diffusivity using DTI. The immediate effects of FUS–BBBD were investigated at 5 min after sonication, and we found significant reductions in ADC and RD values in both sonication groups. A decrease in ADC in sonication regions indicates relatively slower water diffusion arising from restrictions as compared to the water diffusion in the contralateral regions (Sotak, 2004). An early report by Yuan et al. (2017) showed a restriction in water molecules by low-intensity transcranial ultrasound stimulation and suggested that a decrease in ADC signal might be related to the swelling of astrocytes resulting from neuronal excitability caused by ultrasound stimulation. Chu et al. (2014, 2015) found a transient decrease in the ADC signal and suppressed neuromodulation accompanying FUS–BBBD. Thus, we could have a different hypothesis by which cellular swelling leads to a decrease in ADC, which requires further investigation. When we investigated the long-term effects by follow-up measurements at 24 and 48 h, we found an increase in water diffusivity at 24 h in group-0.65 MPa compared to the sham group, although these differences were not statistically significant. We then applied a higher acoustic pressure (0.75-0.80 MPa) to examine water diffusivity under a higher degree of BBBD, and found a statistically significant enhancement in the ADC signal at 48 h. This indicates that water molecules in the sonication region diffuse faster than those in the contralateral region and increase in the volume fraction of the interstitial space of the tissue, accompanied by a large net displacement of water molecules by permeability in the focal region (Sotak, 2004). These results were in accordance with Sepehrband’s study, which showed increased ADC and decreased FA owing to a higher amount of perivascular space fluid (Sepehrband et al., 2019). For further evaluation, we performed analysis of AD and RD signals to calculate diffusivity in the parallel or perpendicular direction to the tract within the voxel of interest, because ADC removed the effects of structural anisotropy by averaging the diffusivity. Accordingly, we found comparable patterns of signal changes in AD and RD compared with ADC. Moreover, we targeted CP and TH by the FUS and DTI measures in these regions did not show significant changes (Supplementary Figures 2, 3). Although there might be anatomical regional differences subject to glymphatic transport (Iliff et al., 2013), a comparable degree of BBBD in both sonication regions is expected to have comparable changes in DTI measures.

The dramatic enhanced ADC signal observed in group-0.80 MPa compared to group-0.65 MPa at 24 and 48 h may be due to critical differences in the biological effects of these stimuli. One possibility is that group-0.80 MPa induced red blood cell extravasation, while group-0.65 MPa did not. Several mechanisms could account for fluid transport, such as physiological water and homeostasis, as well as brain edema (Ohata and Marmarou, 1992; Thrane et al., 2014). Thus, in addition to pressure-driven water transport, pathological features such as transient ischemia and suppression of cortical function by FUS–BBBD (Chu et al., 2015), could result in the presence of fluid in the focal regions by higher sonication. Although safety issues continue to be a concern, we attempted to examine various parameters that cover both physiological and pathological conditions. Further studies are needed, as much remains to be understood about the water dynamics by FUS–BBBD both in normal physiology and during a pathology.

Earlier studies on ultrasound stimulation via DTI measures have shown a reduction in the ADC signal (Schneider et al., 2006; Chu et al., 2014; Yuan et al., 2017). Yuan et al. (2017) reported a restriction in water molecules by low-intensity transcranial ultrasound stimulation. Schneider et al. (2006) reported a dose-dependent damaging effect and a significant decrease in the ADC and an increase in the T2 relaxation time by transcranial low-frequency 20 kHz ultrasound, which infers vasogenic and cytotoxic brain edema. In contrast to previous studies that focused on the application of ultrasound in neuromodulation and thrombolysis, we investigated how FUS–BBBD alters DTI measures, and found a transient decrease in ADC and RD signal changes at 5 min after sonication, while Schneider et al. (2006) reported a significant ADC decline that persisted after 5 days after sonication, indicating cytotoxic edema. Chu et al., investigated structural and functional modulation after FUS–BBBD through ADC and somatosensory evoked potentials. They found a reduction in ADC and suppression of neural activity until 48 h after FUS–BBBD, which infer that FUS–BBBD-induced temporal edema suppressed neural activity (Chu et al., 2014). These results differ from ours, suggesting that, in the previous report, different FUS conditions were imposed, and a link with the clearance system was not introduced. Here, we investigated other diffusion parameters (ADC, AD, RD, and FA) and water channel expression of AQP-4 to elucidate the influence of FUS–BBBD on water dynamics, and found an enhanced magnitude of the diffusion of water molecules. Karakatsani et al. (2020) evaluated the DTI measures as a promising candidate to replace T1-weighted MR images in detecting BBBD and showed a clear FA increase at the site of BBBD. In contrast, we found significant and transient reductions in ADC and RD signals that showed an increase in the mean diffusivity in the area of BBB opening. This is because the target regions had higher FA values without sonication, as shown in Figure 5 in group-sham, which indicated its intrinsic anisotropic structure. Therefore, the different results we found may be explained by the differences in the biological interactions due to BBBD and various conditions of ultrasound sonication, such as frequency, intensity, type of ultrasound (non-focal or focal), and varied detection time of the ADC values.

We assessed water mobility in the local perivascular region through AQP-4 expression using immunohistochemistry, as AQP-4 could provide a unique opportunity to examine the molecular mechanisms underlying water movement (Badaut et al., 2011). We found that AQP-4 expression was upregulated following FUS–BBBD in both sonication conditions at 48 h after BBBD. Recent studies have suggested that the AQP-4 subcellular localization in astrocytes and cell-surface abundance rapidly increases membrane water permeability to prevent CNS edema (Kitchen et al., 2015, 2020). When we performed immunohistochemistry for GFAP and AQP-4, we observed increases in AQP-4 expression and co-localization of AQP-4/GFAP in sonication region after FUS–BBBD (Figure 7). These results demonstrate that FUS–BBBD facilitates water fluid through AQP-4 water channels. As the glymphatic system is a brain-wide network of perivascular pathways that support the exchange of CSF-ISF, studies of the glymphatic system are related to animal experiments with AQP-4 deletion, resulting in impaired CSF influx and Aβ clearance (Iliff et al., 2012; Xu et al., 2015). Although inhibition of AQP-4 activity resulted in decreased diffusivity (Badaut et al., 2011; Debacker et al., 2020), water dynamics in the local perivascular region are yet to be clearly established. Our results suggest that FUS–BBBD can temporarily, locally modulate water fluid transport in normal rats.

On longitudinal observation of DTI measures and AQP-4 staining, significant changes were observed at 48 h in group-0.80 MPa, and this suggested that tissue damages might be the main potential mechanisms for water diffusivity changes by FUS–BBBD. We attempted to conduct AQP-4, H&E, and FJC staining for FUS–BBBD with an intermediate condition of acoustic pressure (0.72 MPa for CP and 0.66 MPa for TH) to examine water diffusivity and tissue damage (Supplementary Figure 4). There was an increase in AQP-4 signal change in the intermediate pressure condition compared to that in group-0.65 MPa and group-sham, but these differences were not statistically significant. For the intermediate condition of acoustic pressure, we found few extravasated red blood cells and damaged neurons, but this was less severe compared to that in group-0.80 MPa. We, therefore, believe that the sonication condition of group-0.65 MPa would be the threshold level of BBBD that may be safe, and it would be highly possible for further experiments of frequent FUS exposures. Thus, further studies covering various parameters are required to investigate pressure-driven water transport during safe FUS exposure.

Diffusion tensor imaging is widely used for clinical diagnosis, and changes in ADC have been correlated with changes in AQP-4 expression under normal and pathological conditions (Tourdias et al., 2009; Badaut et al., 2011). Accordingly, we evaluated the relationship between the ADC and AQP-4 (Supplementary Figure 5). We found that the FUS–BBBD altered the AQP-4 and ADC levels, where the signal changes in ADC and AQP-4 expression were positively correlated. Although we observed a significant correlation between the ADC and AQP-4, different patterns of decreased ADC with upregulated AQP-4 were observed at 5 min in group-0.65 MPa; the decreased ADC may be regarded as transient cell swelling through changes in the local blood flow, and no significant upregulation of AQP-4 expression may have been caused by increased BBB permeability. The precise mechanisms of FUS-induced BBBD that influence astrocytes to modulate local water transformation are still unknown. Thus, we planned to conduct more experiments to address the effects of FUS-induced BBBD on water clearance, as we considered a relatively small sample size for the evaluation of the immunohistochemistry. Although further experimental verification is required, this study is novel because, to the best of our knowledge, it is the first to report on the relationship between ADC and AQP-4 expression through FUS–BBBD in the normal brain, whereas existing studies have focused on pathophysiological situations.

The DTI parameters are sensitive to pathological processes and are thus widely used in many clinical applications (Dong et al., 2004; Alexander et al., 2007; Lerner et al., 2014), while a host of research has reported an increase in the ADC and a decrease in the FA in pathological conditions. Here, we found that the slight increase in ADC signal at 24 h was restored to the sham condition level at 48 h in group-0.65 MPa; however, this was not statistically significant. Group-0.80 MPa showed a consistent increase in ADC until 48 h owing to the higher sonication conditions. We performed a follow-up DTI until 72 h after BBBD with higher FUS conditions (Figure 5) to determine whether this enhancement of the ADC was permanent or not. The ADC value was restored to a value relative to that of the contralateral region at 72 h. These results suggest possible edema, which is transient and reversible, could have been caused by the high sonication conditions, and was cleared over the course of 72 h. Furthermore, we found a consistent decrease in FA, which could be pathological. However, the diffusion of water through the BBB increased diffusivity, which may have affected the decrease in FA. A recent report suggested that a low FA is also related to swollen astrocytes stained with AQP4 and GFAP after neonatal hypoxia-ischemia (HI) (Lee et al., 2021). In this study, we observed that group-0.8 MPa induced significant decreases in the FA signal at 24 h and 48 h (Figure 5). The expression of AQP4 and swollen astrocyte was also increased in the Group-0.8 MPa at 48 h after FUS–BBBD (Supplementary Figure 4). These results suggest that increased AQP4 and swollen astrocytes may be related to the decrease in FA value reflecting the 0.8 MPa BBBD condition mimicking pathological conditions. To evaluate the relation of isotropic diffusion with ADC and FA, the correlation between ADC and FA was calculated (Supplementary Figure 6), indicating significant negative correlations (*r* = 0.925, *p* < 0.05). In addition, Pasternak et al. (2009) proposed a free water elimination algorithm by modifying the imaging sequence and found an intact fiber structure where FA decreased. Sepehrband et al. (2019) reported that perivascular space fluid affects DTI-derived measures, resulting in an increased ADC and decreased FA when the perivascular space increases. These results imply that water transport through the BBBD could be a major contributor to DTI-derived measures.

This study has several limitations. The sample size was relatively small for the individual experimental groups, and more experiments could yield improved estimates of local water dynamics. To clarity the localization and functionality for AQP-4 along with perivascular region, double staining with a vascular marker such as CD31 or collagen IV will be needed for further experiments. Here, we found mild hemorrhagic foci at the center of the ultrasound focus in group-0.80 MPa, and thus use of different techniques to evaluate the damaging effects, such as immunofluorescence data on gliosis marker and immunohistochemistry, should be performed to confirm precise mechanisms of these findings. Long-term follow-up analysis in group-0.80 MPa will be important to determine water dynamics in relation to pathology and functional outcomes.

## Conclusion

This study demonstrated, for the first time, the local dynamics of water molecules caused by FUS-induced BBBD using different DTI parameters and water channel expression of AQP-4 in the normal brain. Two different sonication conditions (group-0.65 MPa and group-0.80 MPa) revealed consistent patterns of water transport resulting in a transient decrease and subsequent increase in the ADC and upregulation of the AQP-4 expression. Our results may improve the molecular understanding of the underlying mechanisms of FUS-induced BBBD as a therapeutic tool.

## Data Availability Statement

The original contributions presented in the study are included in the article/Supplementary Material. Further inquiries can be directed to the corresponding author.

## Ethics Statement

The animal study was reviewed and approved by the Daegu Gyeongbuk Medical Innovation Foundation (DGMIF) Institutional Animal Care and Use Committee (IACUC). Approval number: DGMIF-19100701-00.

## Author Contributions

MH and JP conceptualized and designed the experiments. MH and HC performed the MRgFUS experiment and analyzed the MR data. HC and E-HL performed and evaluated the immunohistochemistry. MH and HS prepared the manuscript. JP edited and revised the manuscript. All authors contributed to the article and approved the submitted version.

## Conflict of Interest

The authors declare that the research was conducted in the absence of any commercial or financial relationships that could be construed as a potential conflict of interest.

## Publisher’s Note

All claims expressed in this article are solely those of the authors and do not necessarily represent those of their affiliated organizations, or those of the publisher, the editors and the reviewers. Any product that may be evaluated in this article, or claim that may be made by its manufacturer, is not guaranteed or endorsed by the publisher.
