# Supplementary material for: Localized Modification of Water Molecule Transport After Focused Ultrasound-Induced Blood–Brain Barrier Disruption in Rat Brain
Source: Front Neurosci. 2021 Jul 29;15:685977. doi: 10.3389/fnins.2021.685977 (PMC8358078; doi:10.3389/fnins.2021.685977)
Supplement: Supplementary file 1 [file Data_Sheet_1.pdf]

frontiers

Localized Modification of Water Molecule Transport after Focused Ultrasound-induced Blood-Brain Barrier Disruption in Rat Brain

Mun Han[1†], Hyeon Seo[1†], HyoJin Choi[1], Eun-Hee Lee[1], and Juyoung Park[1*]

[1]Medical Device Development Center, Daegu-Gyeongbuk Medical Innovation Foundation, Daegu, Korea

*** Correspondence:**
Juyoung Park
jyp@dgmif.re.kr

[†] These authors contributed equally to this work

Supplementary Figures

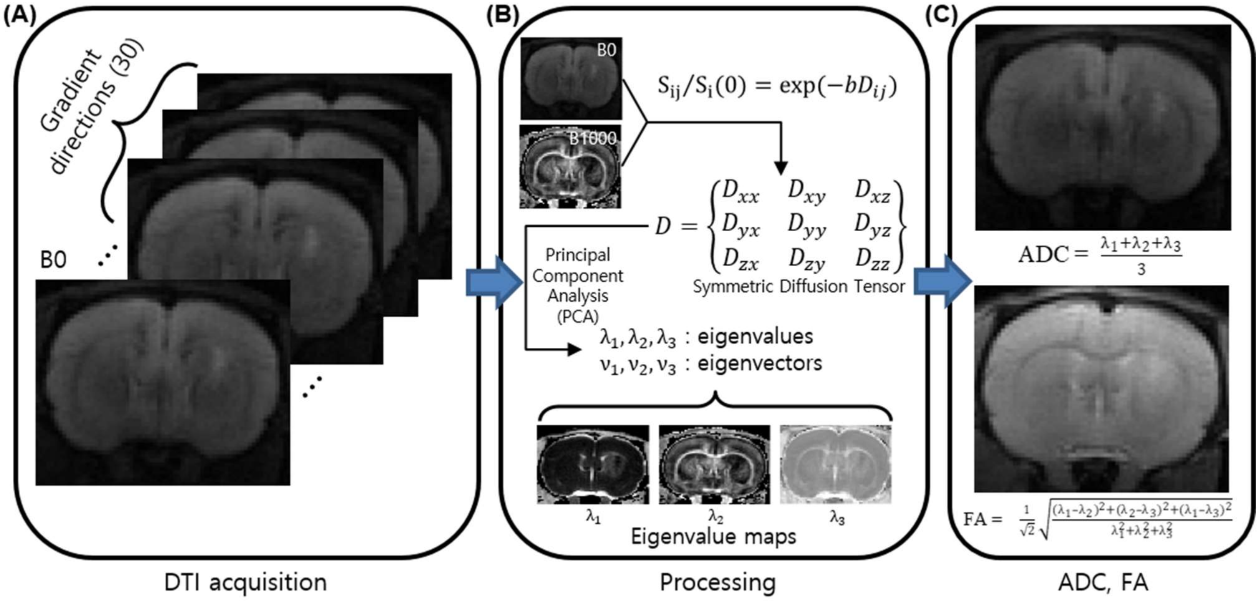

Supplementary Figure S1. Schematic diagram of the acquisition and processing of DTI. The DTI was acquired with 30 different diffusion gradient directions (A), and we process three diffusion eigenvalues (B) to calculate the ADC and FA (C).

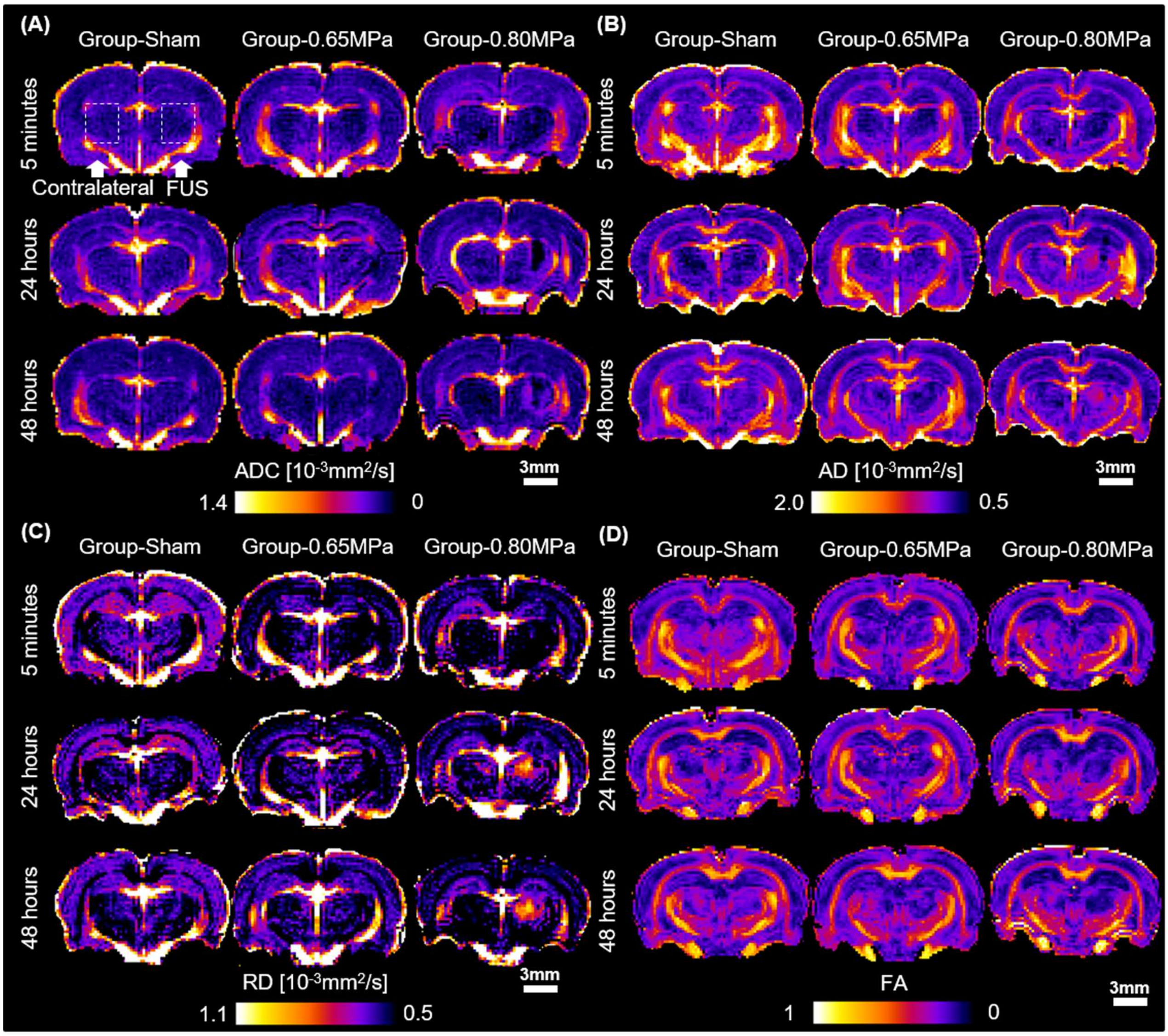

Supplementary Figure S2. DTI measures after FUS–BBBD. (A) Representative maps of apparent diffusion coefficient (ADC), (B) axial diffusivity (AD), (C) radial diffusivity (RD), and (D) fractional anisotropy (FA) at thalamus. Scale bar: 3 mm.

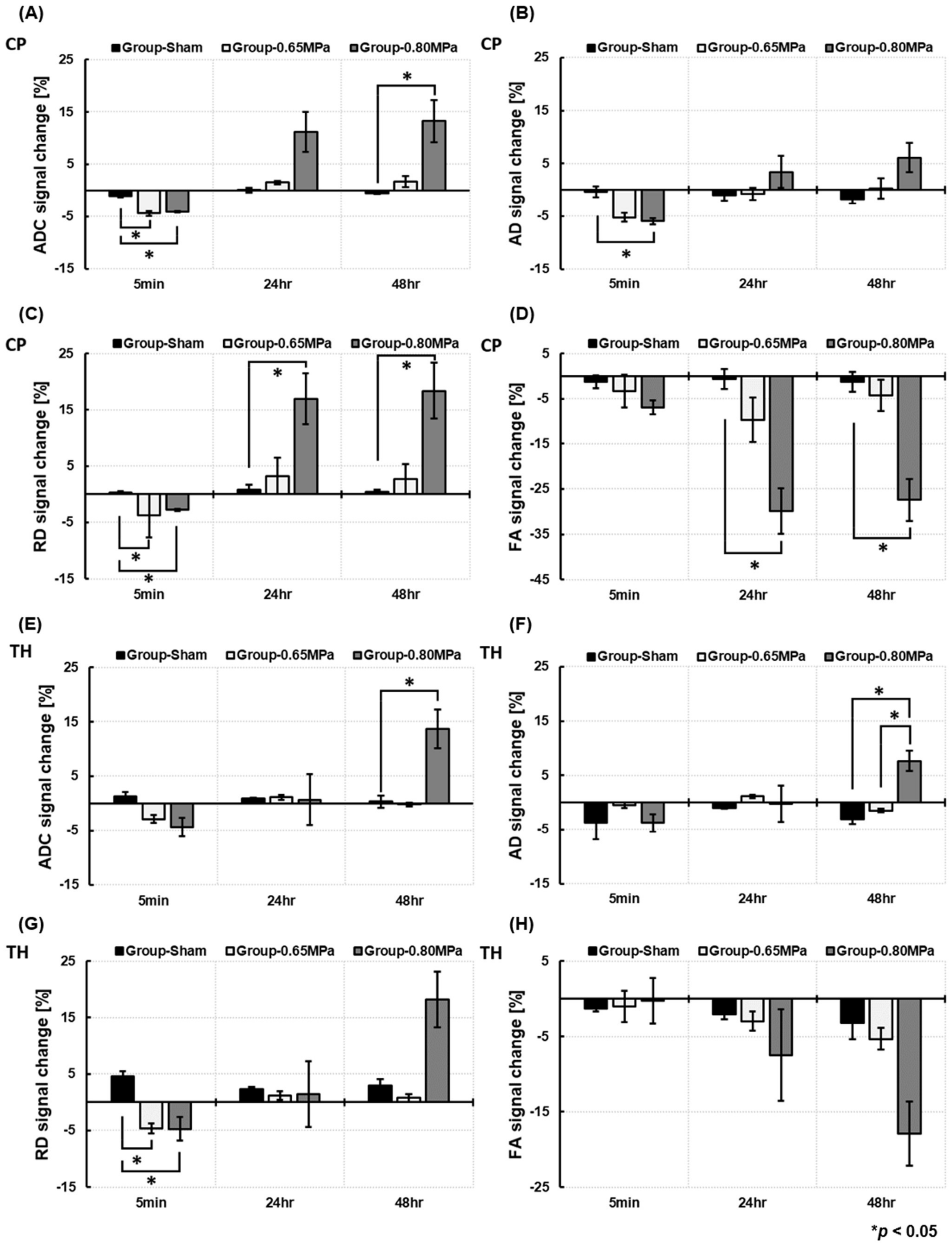

(A)
CP
Group-Sham
Group-0.65MPa
Group-0.80MPa
ADC signal change [%]
5min
24hr
48hr
(B)
CP
AD signal change [%]
(C)
CP
RD signal change [%]
(D)
CP
FA signal change [%]
(E)
TH
ADC signal change [%]
(F)
TH
AD signal change [%]
(G)
TH
RD signal change [%]
(H)
TH
FA signal change [%]
*p < 0.05

Supplementary Figure S3. Comparison of DTI measures (ADC (A and E), AD (B and F), RD (C and G), and FA (D and H)) based on the signal change ratio between group-sham, group-0.65 MPa, and group-0.80 MPa after 5 min, 24h, and 48h after FUS-BBBD at caudate putamen (A-D) or thalamus (E-H).

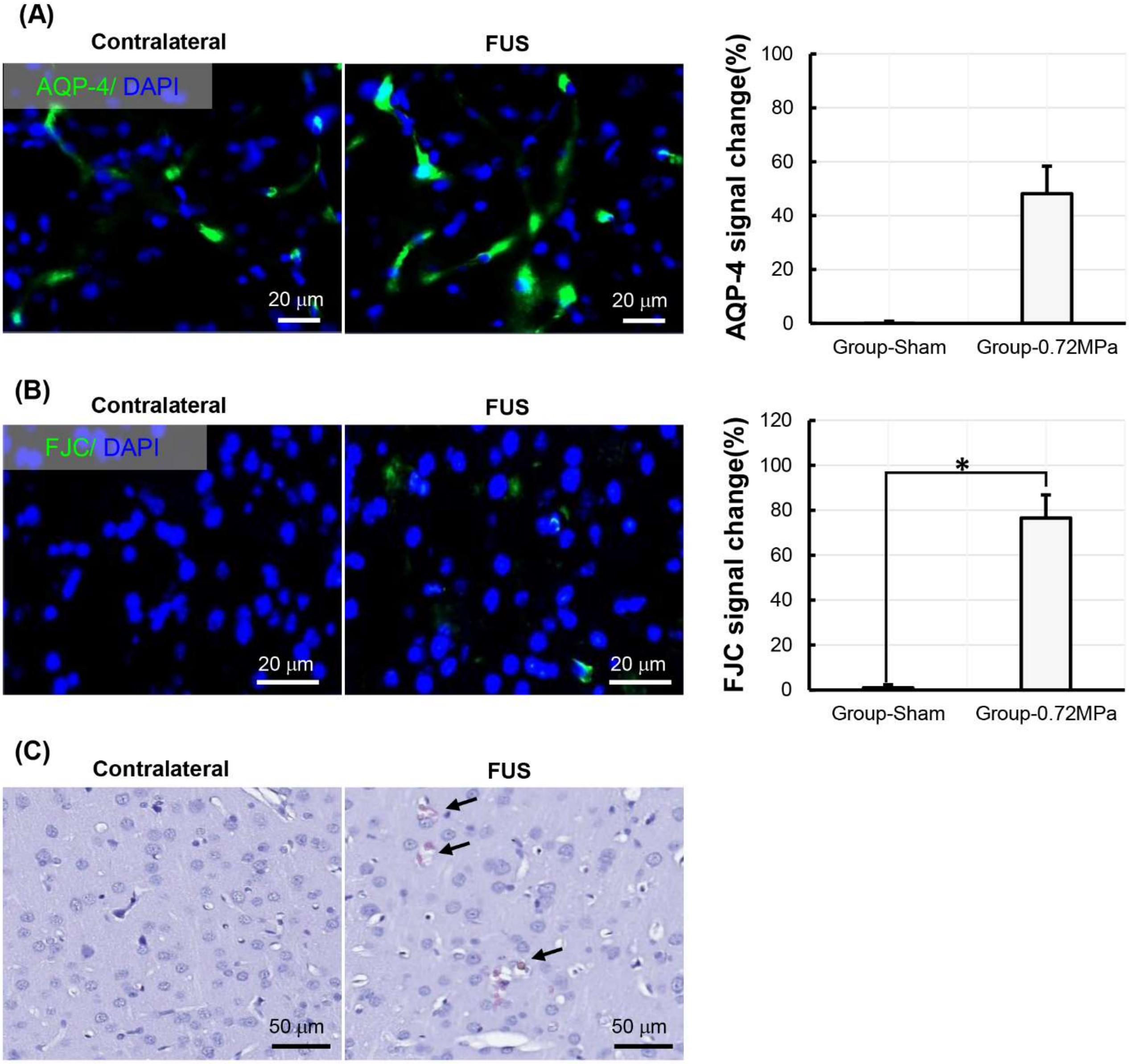

Supplementary Figure S4. Representative AQP-4 immunostaining (A), FJC histology (B), and H&E histology (C) of the caudate putamen in the intermediate condition of acoustic pressure. We applied a pressure amplitude of 0.72 MPa for the caudate putamen and 0.66 MPa for the thalamus, and the brain section was stained at 48 h after FUS–BBBD. Quantification of AQP-4 expression and FJC histology is analyzed compared to that of group-sham. *$p < 0.05$.

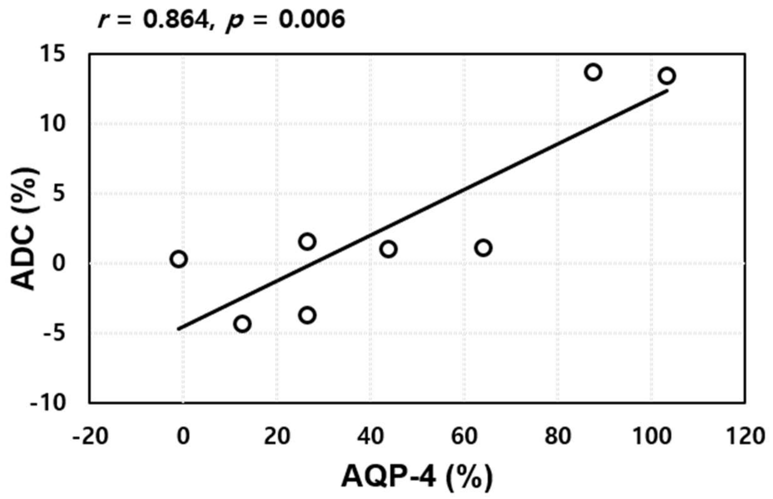

Supplementary Figure S5. Correlation analysis between ADC and AQP-4 signal changes. The ADC and AQP-4 signal changes were acquired at 5 min, 24 h, 48 h in group-0.65 MPa and at 48 h in group-0.80 MPa. Relationship between the ratio of ADC was positively correlated with that of the AQP-4 expression (r = 0.864, p = 0.006, Pearson's correlation).

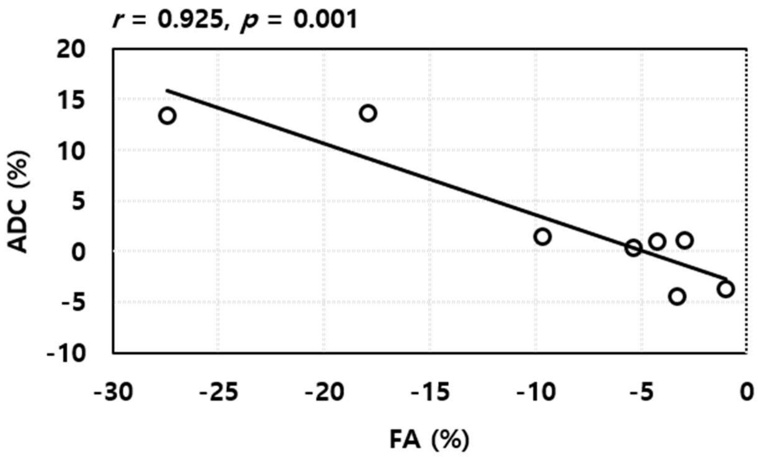

Supplementary Figure S6. Correlation analysis between ADC and FA. Relationship between the ratio of ADC was negatively correlated with that of the FA ($r = 0.925$, $p = 0.001$, Pearson's correlation).
